# ANN-based prediction of torsional fatigue behavior in an injection-molded compliant mechanism component

**DOI:** 10.1371/journal.pone.0361201

**Published:** 2026-10-28

**Authors:** Vu Quang Huy, Tran Minh The Uyen, Pham Son Minh, Van-Thuc Nguyen, Hai Nguyen Le Dang

**Affiliations:** 1 Faculty of Advanced Education, Ho Chi Minh City University of Technology and Engineering, Ho Chi Minh City, Vietnam; 2 Faculty of Mechanical Engineering, Ho Chi Minh City University of Technology and Engineering, Ho Chi Minh City, Vietnam; University of Vigo, SPAIN

## Abstract

An Artificial Neural Network (ANN) model was created to predict how injection-molded parts made from flexible mechanisms would hold up under twisting fatigue. These parts were made from three common plastics—ABS (Acrylonitrile Butadiene Styrene), PP (polypropylene), and PA6 (Polyamide 6)—strengthened with up to 30 wt.% short glass fibers. The team also looked at how many times the parts were cycled during manufacturing.To test them, the samples were twisted back and forth ±40° for 100 cycles. The study examined how the type of plastic, the amount of glass fiber, and key injection molding settings affected the results. It turned out that PA6’s ability to withstand fatigue really improved when it had more glass fiber, especially 20 wt.% or more. PA6 turned out to be the strongest material in this test, followed by ABS. PP was the most consistent but also the weakest in terms of twisting strength. The ANN model was quite successful at predicting the twisting force, with a match range (R ≈ 0.94 to 0.98) and cross validation 0.93 and 0.97. The research also pointed out specific injection molding conditions—like melt temperature, filling pressure, and packing pressure—as being particularly important for the final performance. Overall, this proposed approach offers a tool to help engineers design and make plastic flexible mechanisms more effectively.

## 1. Introduction

Compliant mechanisms, which use elastic deformation to achieve motion as opposed to the discrete motion of joint-based mechanisms, have been of interest for robotic and industrial applications due to their simplicity and compactness while reducing maintenance needs [1–4]. The inherent compliant nature of these mechanisms creates difficulties in achieving accurate force or torque control, especially in applications involving constant torque over various deflection angles in a torsional motion scenario. This is primarily due to the low torsional stiffness of these mechanisms, which is a major factor in determining the stability of the mechanism’s performance [5–7]. It is therefore necessary to develop techniques to decouple the effects of low torsional stiffness from constant torque control.

In terms of various parameters affecting the performance of compliant mechanisms, the geometric configuration of flexural elements is critical to determining the performance of the mechanism in terms of achieving constant torque while maintaining low stiffness values in the torsional direction of motion. This paper aims to focus on the effects of variations in the geometric configurations of compliant mechanism parts designed for injection molding and their influence on the torsional strength of these mechanisms while achieving constant torque values. The aim is to develop a relationship between geometric configurations and their effects on the mechanical responses of these mechanisms to achieve tunability in terms of stiffness values and optimize the design of these mechanisms for various applications [8,9].

Geometric configurations have been proven to be effective in achieving various effects on stress values, deformation responses, and mechanical efficiency of these mechanisms and their responses to various internal forces and deformations [10,11]. Previous studies have proven that optimized internal geometries of these mechanisms can be used to improve their load capacity and achieve variable stiffness values for various applications and operating conditions [12–15]. These properties are critical to achieving variable stiffness values and efficiency for mechanisms designed to operate within the parameters of polymer-based compliant mechanisms and manufacturing techniques.

In addition, the ability of polymer-based components, when subjected to cyclic loading, has been a significant concern, particularly when components are subjected to torsional loading, as observed in compliant mechanisms. Fatigue response is one of the critical factors affecting the overall reliability of components subjected to torsional loading. Fatigue response has been a significant concern, and conventional methods of fatigue prediction, although accurate, have been found to be ineffective when dealing with components subjected to injection molding, where anisotropic and stress concentrations are often encountered [16,17].

In order to overcome the challenges encountered when dealing with conventional methods of fatigue response, artificial neural networks have been identified as an accurate tool for predicting nonlinear response, as observed in fatigue analysis. ANNs have been identified as an accurate tool for predicting fatigue response, allowing accurate fatigue response prediction through learning from experimental data, thus eliminating the need for expensive testing methods [18]. Fatigue response has been identified as an accurate tool for predicting fatigue response, particularly when dealing with composite materials, as observed in glass fiber-reinforced composites [19,20]. In addition, ANNs have been identified as accurate tools for predicting various properties of composites, particularly when subjected to various testing conditions, thus providing significant advantages when dealing with composites [21]. But they have several well-known limitations, such as: requiring large datasets, dependence on data quality

In consideration of the challenges encountered when dealing with conventional methods of fatigue response, this study aims to develop an accurate tool for predicting fatigue response, particularly when dealing with constant moment compliant mechanism components subjected to torsional loading, using ABS, PP, and PA6, with various glass fiber reinforcement, from 0–30 wt.%.

## 2. Materials and methods

### 2.1. Material of compliant mechanism design

The compliant mechanism [22] depicted in Fig 1, which is the focus of this study, was cleverly engineered to transfer rotational torque by using elastic deformation instead of the typical rigid joints found in traditional designs. This structure is made up of a circular outer ring that measures 100 mm in diameter and 5 mm in thickness. This ring is linked to a central hub by four flexible beams, each 1 mm thick, that are evenly spaced around the circle. These beams are the compliant elements that let the structure deform in a controlled way when twisted. The way these beams are arranged symmetrically helps to spread out the stress evenly and ensures the mechanism behaves reliably while it’s being used. The outer ring acts like a sturdy support, and the central hub is where the twisting force, or torque, is applied. When a twisting load is put on the mechanism, the flexible beams bend elastically and store some energy in the form of strain. This smart design allows for smooth movement to be passed along with less friction and fewer parts compared to other mechanical setups.

**Fig 1:**
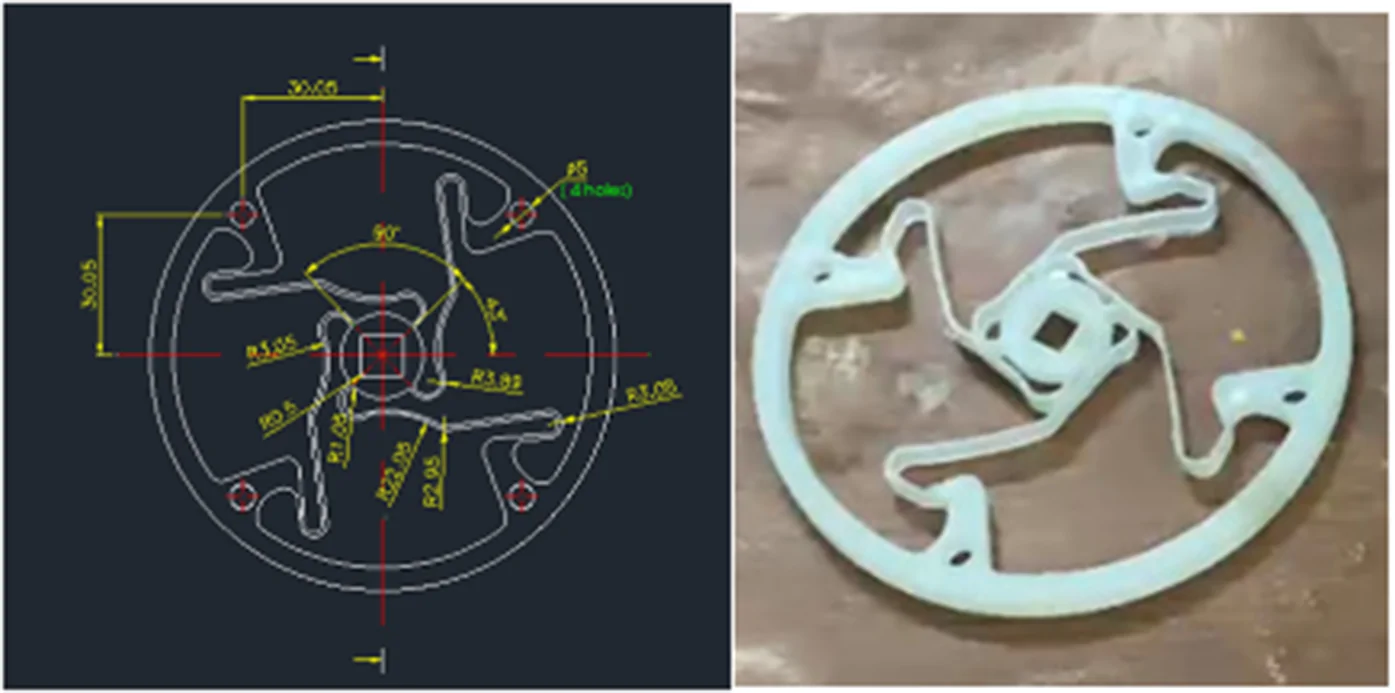
(a) 2D sketch of the compliant mechanism (b) 3D model of the compliant mechanism.

The parts for the equal moment compliant mechanisms were made using a plastic injection molding process. We used three different types of thermoplastic materials: Acrylonitrile Butadiene Styrene (ABS), Polypropylene (PP), and Polyamide 6 (PA6). These materials came in both their standard form and a reinforced version that included glass fibers. The main goal was to carefully change the amount of glass fiber in the PA6 to see how it affected the material’s performance under twisting and repeated stress.

Before the molding process began, the plastic granules were dried according to the suggested guidelines – 80 °C for the ABS and PP types, and 110 °C for the PA6 type – with the drying lasting between 2 and 4 hours. This step was taken to reduce any potential problems caused by moisture.

The specific design being made – a circular flexure mechanism that resists bending moments, featuring four flexible beams (each 1 mm thick) linking a central part to an outer ring measuring 100 mm in diameter and 5 mm thick – was created using a multi-cavity mold that was already available.

Key settings for the injection molding machine, like the temperature of the molten plastic, the pressure used to pack it in, how long that pressure was applied, and the pressure during filling, were all adjusted. They were set within ranges suitable for each material to make sure the mold filled completely and to keep internal stresses low. However, certain parameters were deliberately changed to produce different internal structures in the parts, which would later be tested for fatigue. After they were made, the finished pieces were left to sit at room temperature until it was time for testing. The exact mix and amount of the plastic materials used were carefully controlled, as detailed in Table 1

**Table 1:** Mixing ratios and mass for each case using PA6 resin.

Percentage	PA6 0 wt.% GF	PA6 30 wt.% GF
TH 0%	700 g	0 g
TH 5%	588.9 g	111.1 g
TH 10%	487.9 g	212.1 g
TH 15%	395.7 g	304.3 g
TH 20%	311.1 g	388.9 g
TH 25%	233.3 g	466.7 g
TH 30%	0 g	700 g


$$\begin{array}{c} \frac{Z}{\text{1}\text{0}\text{0}}=\frac{\text{0}.\text{3}xY}{\text{0}.\text{7}xY+X} \end{array}$$ (1)

With:

X: PA6 + 0 wt.% GF

Y: PA6 + 30 wt.% GF

Z: mass (g)

GF: Glass Fiber

### 2.2. Injection molding

All samples were molded using a Haitian MA 1200 III horizontal injection molding machine (120-ton clamping force). Injection Molding Parameters included filling pressure (55–105 bar), speed (90%), time (2 s), packing pressure (51–93 bar), speed (90%), and time (1 s). PA6 with higher GF required adjusted pressures for flowability. A pre-existing two-plate mold, custom-machined for this specific geometry, was used. The processing parameters were optimized for each material to produce defect-free parts and are summarized in Table 2.

**Table 2:** Optimized injection molding parameters for each material type.

Parameter	ABS	PP	PA6 (0–30 wt.% GF)
Melt Temperature	220 °C	220 °C	280 °C
Filling Pressure	95 Bar	55 Bar	100 Bar
Filling Speed	90	90	90
Packing Pressure	87 Bar	51 Bar	88-93 Bar
Filling Time	2 s	1 s	2 s
Packing Time	1 s	1 s	1 s

Numerous parameters that impact the product’s chemical and physical characteristics must be specified during injection molding. Drying temperature: The plastic particles must be dried before the product is molded. For ABS and PP plastic, the drying temperature is typically 80°C, but for PA6 plastic, it is 110°C. Typically, drying takes two to four hours. Many preliminary tests are carried out to guarantee a successful injection operation, such as filling and a stable shape, before determining the injection settings. Table 3 lists the parameters that must be established during the injection molding process. To ensure optimal filling conditions, the filling pressure and packing pressure are raised in comparison to the pure PA6 case due to the extra fiber-glass.

**Table 3:** Injection parameters.

Material	Filling press (bar)	Filling speed (at % filling press.)	Filling time (s)	Packing press (bar)	Packing speed (at % filling pres.)	Temperature Max. temp: (°C)	Temperature Min. temp: (°C)
PP	55	90	2	51	90	220	200
ABS	95	90	2	87	90	210	190
Pa6 (0 wt.%)	100	90	2	88	90	280	260
Pa6 (5 wt.%)	100	90	2	88	90	280	260
Pa6 (10 wt.%)	100	90	2	88	90	280	260
Pa6 (15 wt.%)	105	90	2	93	90	280	260
Pa6 (20 wt.%)	105	90	2	93	90	280	260
Pa6 (25 wt.%)	105	90	2	93	90	280	260
Pa6 (30 wt.%)	105	90	2	93	90	280	260

### 2.3. Torsional fatigue testing

The lab-built torsional testing machine [23] is designed to evaluate the fatigue behavior of plastic specimens under cyclic torsional loading (Fig 2). The system consists of an 40 W AC electric motor, a belt transmission mechanism, and a 1: 50 reduction gearbox to generate controlled rotational motion. The specimen is clamped between a fixed fixture and a rotating chuck, allowing the applied torque to be transmitted to the test sample. A torque sensor is integrated into the system to measure the torsional moment during operation, while the control and data acquisition are managed through a PLC and monitored using TIA Portal software. This setup enables continuous cyclic loading and accurate measurement of the fatigue performance of the tested specimens.

**Fig 2:**
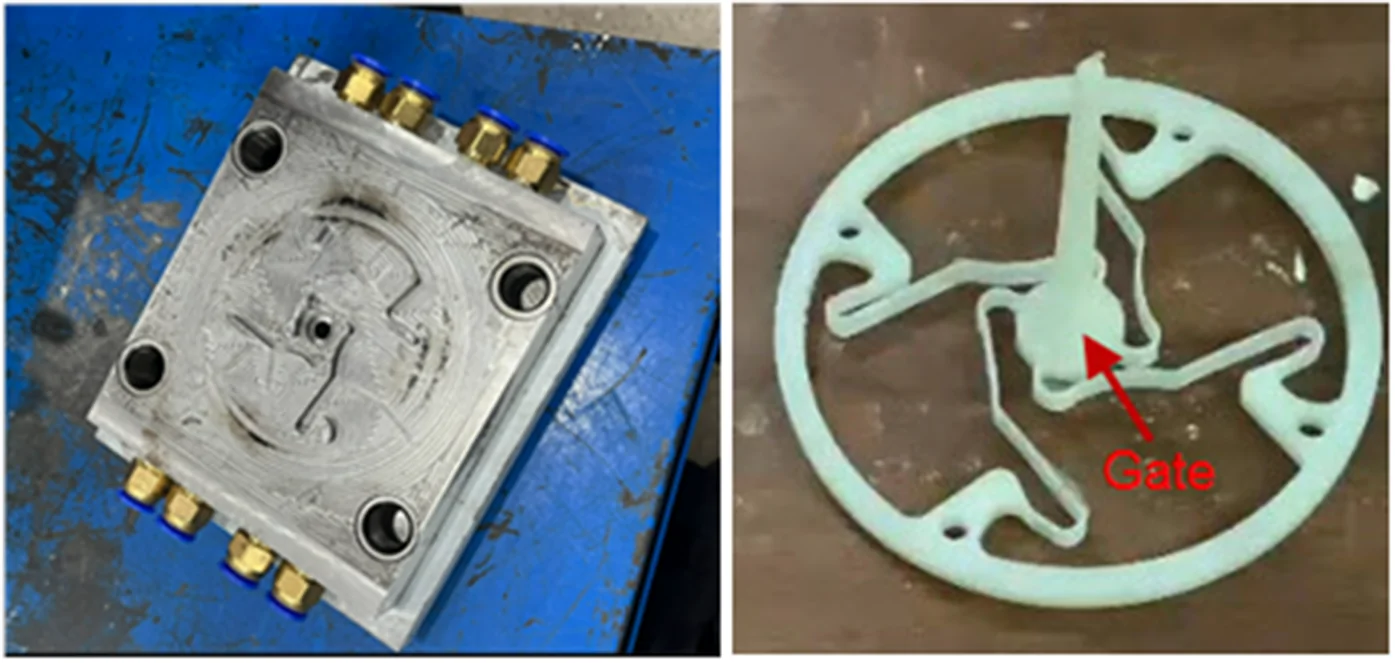
Photo of mold and sample.

The machine in Fig 3 consists of a fixed grip and a rotating grip. One side of the compliant mechanism (the outer ring) was clamped to the fixed grip, which is connected to a torque sensor (0–300 N.m range, accuracy: ± 0.1 N.m). The other side (the central hub) was attached to the rotating grip.

**Fig 3:**
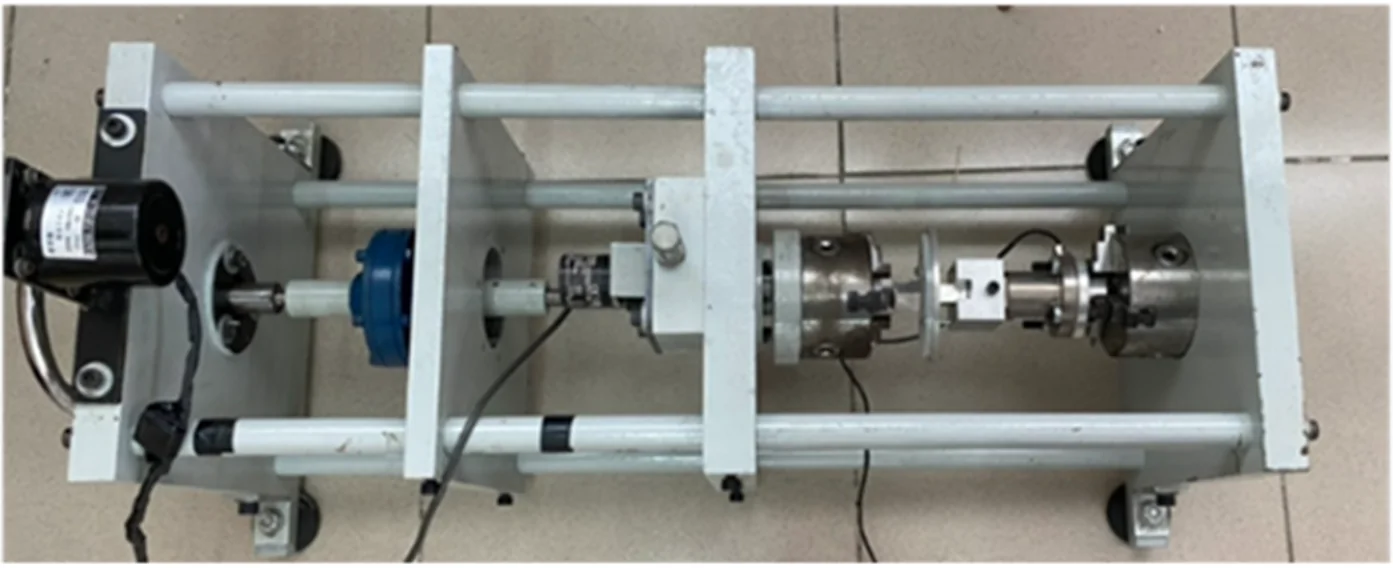
Schematic and photograph of the custom torsional testing setup.

The test protocol involved subjecting each sample to a cyclic angular displacement of ±40 degrees from its neutral position. The machine cyclically rotated the hub, while the torque sensor recorded the resisting moment from the mechanism’s flexible arms. Data was collected for 100 cycles for each sample at ±40° left and +100° right torsion at 45° per min.

Data from the torque sensor and a rotary encoder (for angular position) were acquired via a Siemens PLC system and logged using TIA Portal software. This provided a high-resolution dataset of torque versus cycle number for each experiment.

### 2.4. Artificial Neural Network (ANN) modeling

Artificial neural networks (ANNs) modeling is biologically inspired computer programs designed to simulate the way in which the human brain processes information. ANNs gather their knowledge by detecting the patterns and relationships in data and learn (or are trained) through experience, not from programming. An ANN is formed from hundreds of single units, artificial neurons or processing elements (PE), connected with coefficients (weights), which constitute the neural structure and are organised in layers. Currrent ANN model in Fig 4 has an Input (Filling Speed, Filling time, Packing Speed, Temperature, Tmold, Packing Press), hidden layer (1), Output (Torsional moment). The ANN model employed the hyperbolic tangent sigmoid (tansig) activation function in the hidden layer to capture the nonlinear relationship between the input and output variables, while a linear activation function (Purelin) was adopted in the output layer to predict continuous response variables. The available dataset was randomly divided into three subsets: 70% for network training, 15% for validation, and 15% for testing. The training subset was used to optimize the network weights and biases, the validation subset was employed to monitor the generalization capability and prevent overfitting through early stopping, and the testing subset was used to independently evaluate the predictive performance of the developed ANN model.

**Fig 4:**
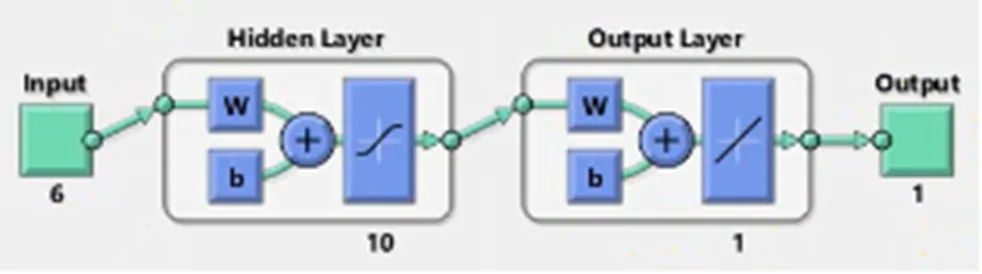
Structure of ANN model.

An Artificial Neural Network (ANN) was developed to predict fatigue behavior. The network was a feed-forward model trained using the experimental results. The inputs to the model included material type and cycle number, while the output was the predicted peak torque. The dataset was partitioned for training, validation, and testing to ensure the model’s generalizability.

## 3. Results and discussion

Local fiber orientation and weld-line formation may contribute to stress concentration and fatigue crack initiation, however we skip weld-line characterization in this research.

The impact of various polymer kinds on the torsional moment is covered in this section. First, tests are conducted on the injected pure ABS material. Next, various polymer types, including PP and PA6, are injected and analyzed. To help show the torsional properties of the injected Compliant Mechanisms, the original torsional moment, its division into positive and negative diagrams, and some comparisons are described.

### 3.1. ANN model validation

The trained ANN model was used to predict the torque-cycle curves for all tested materials. The predictions were then plotted against the actual experimental data. Fig 5–13 show plots of this comparison for ABS, PP, PA6 0 wt.%, 5 wt.%, 10 wt.%, 15 wt.%, 20 wt.%, 25 wt.%, and 30 wt.%.

**Fig 5:**
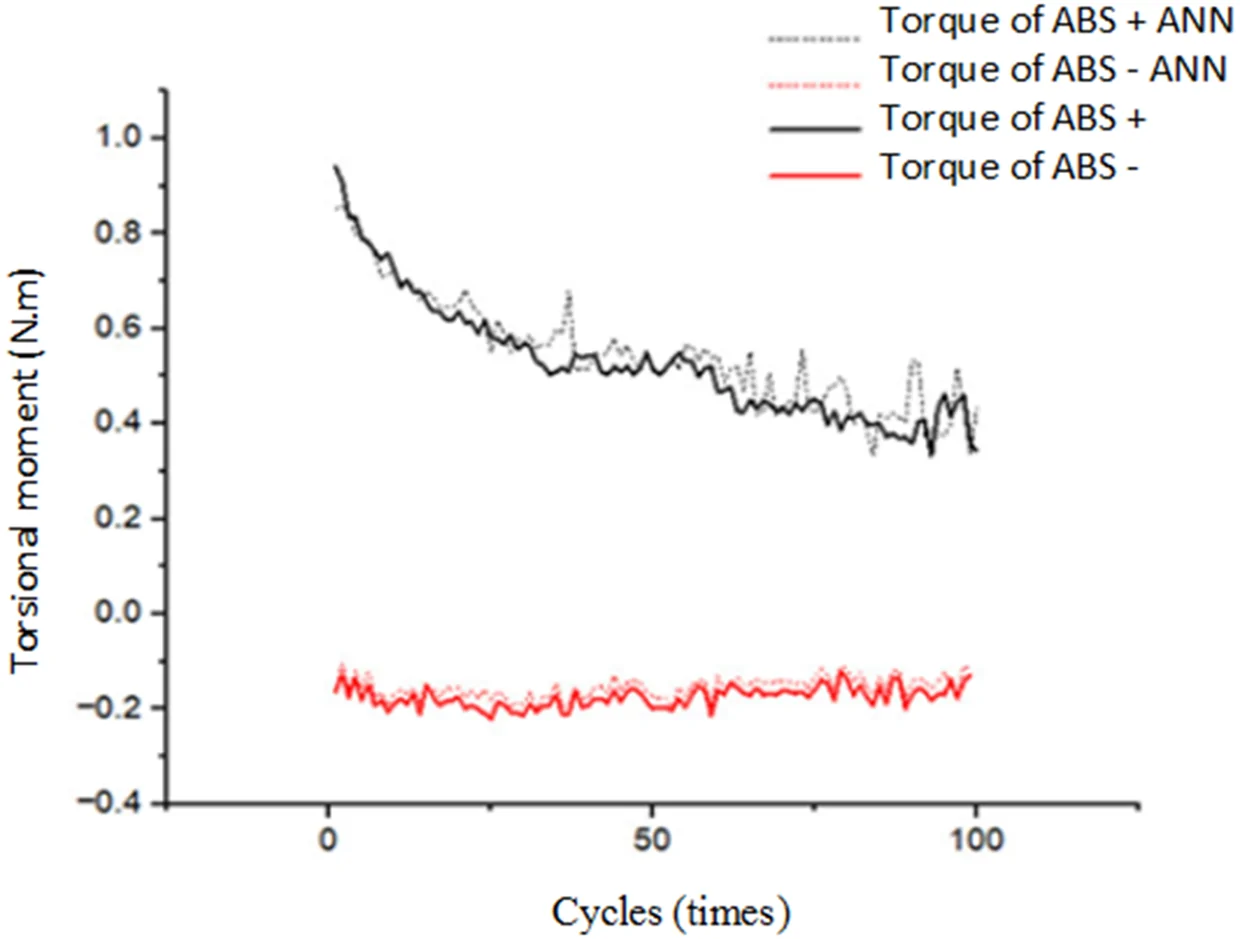
Comparison of experimental data and ANN predictions for ABS.

**Fig 6:**
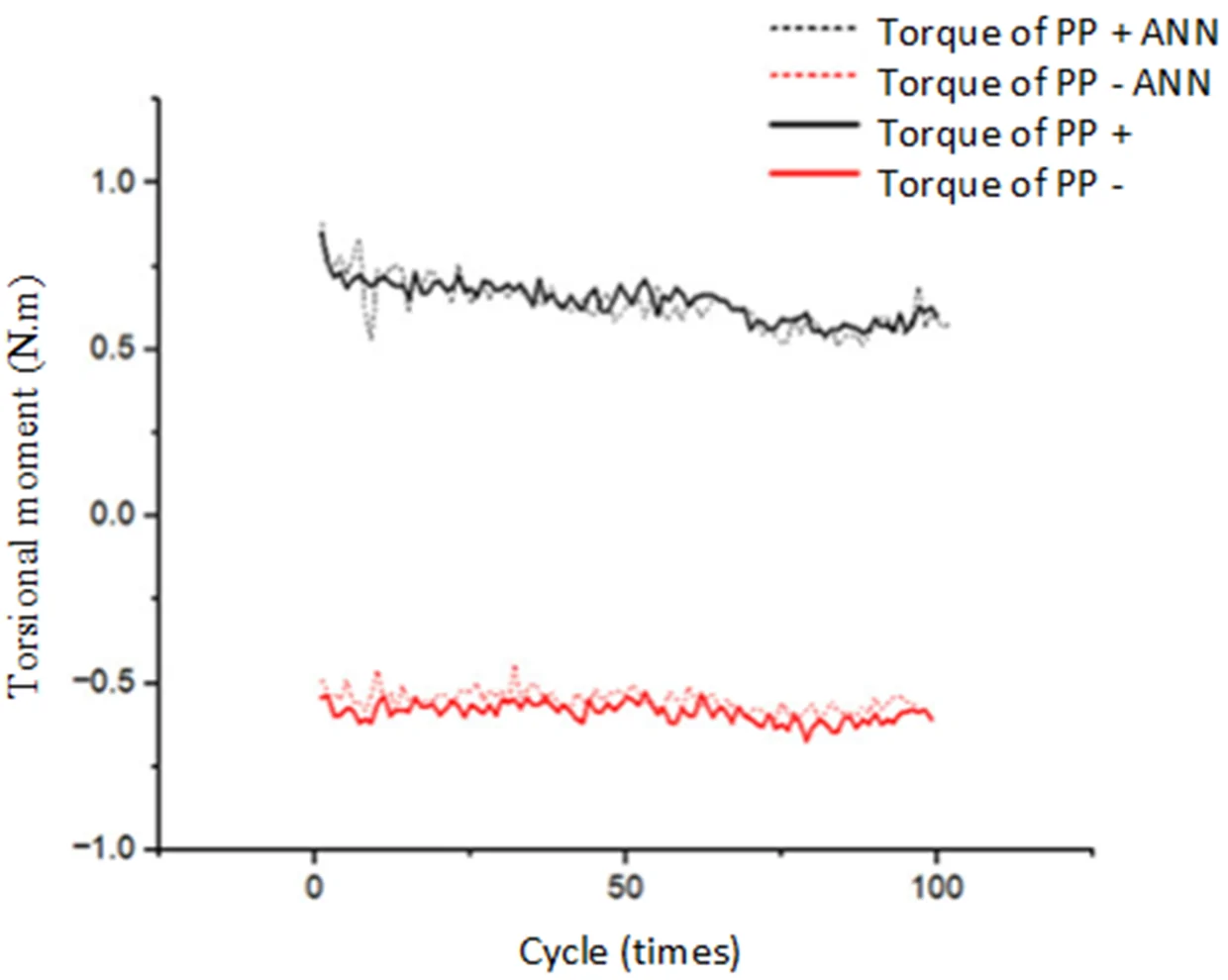
Comparison of experimental data and ANN predictions for PP.

**Fig 7:**
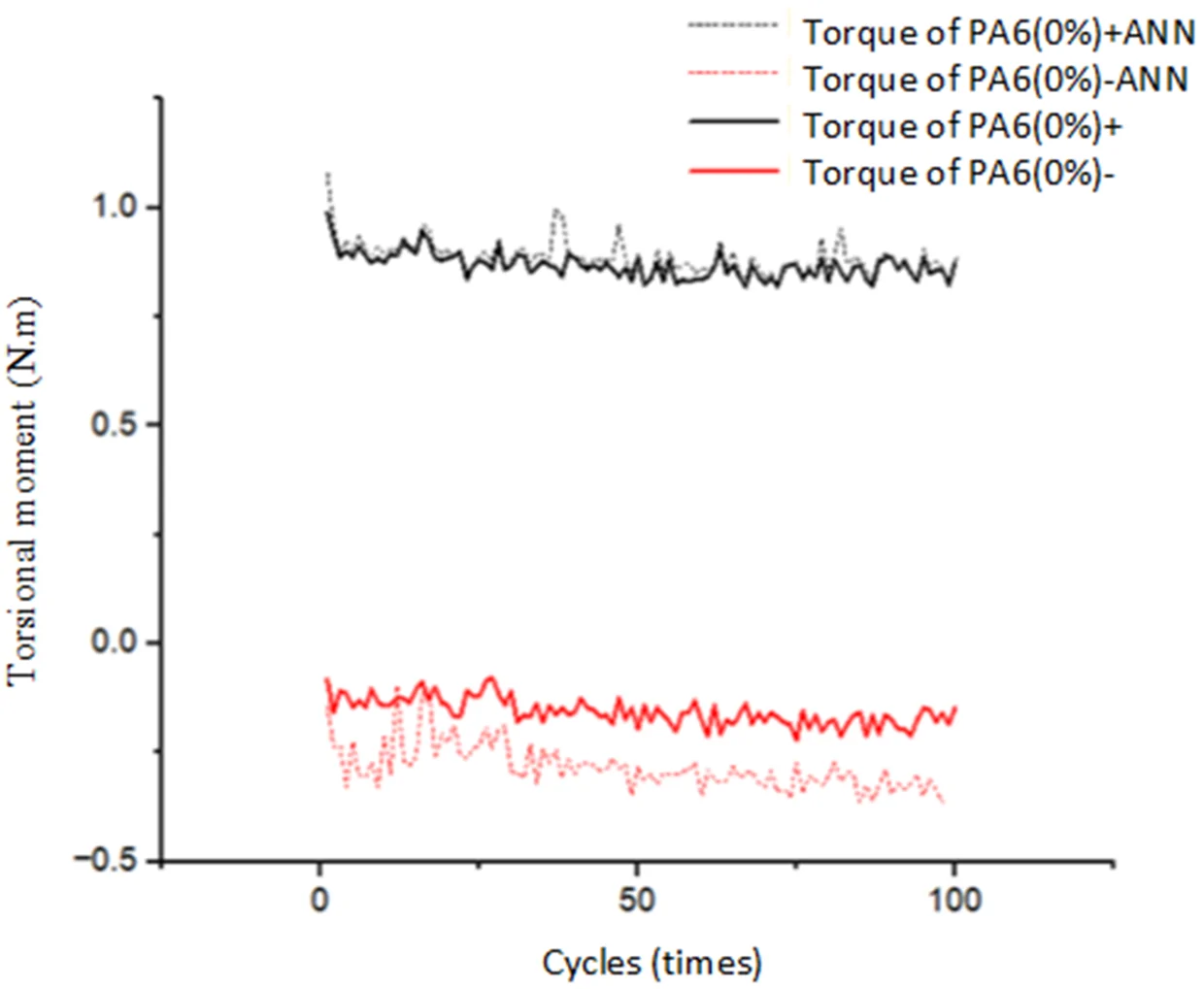
Comparison of experimental data and ANN predictions for PA6 (0%).

**Fig 8:**
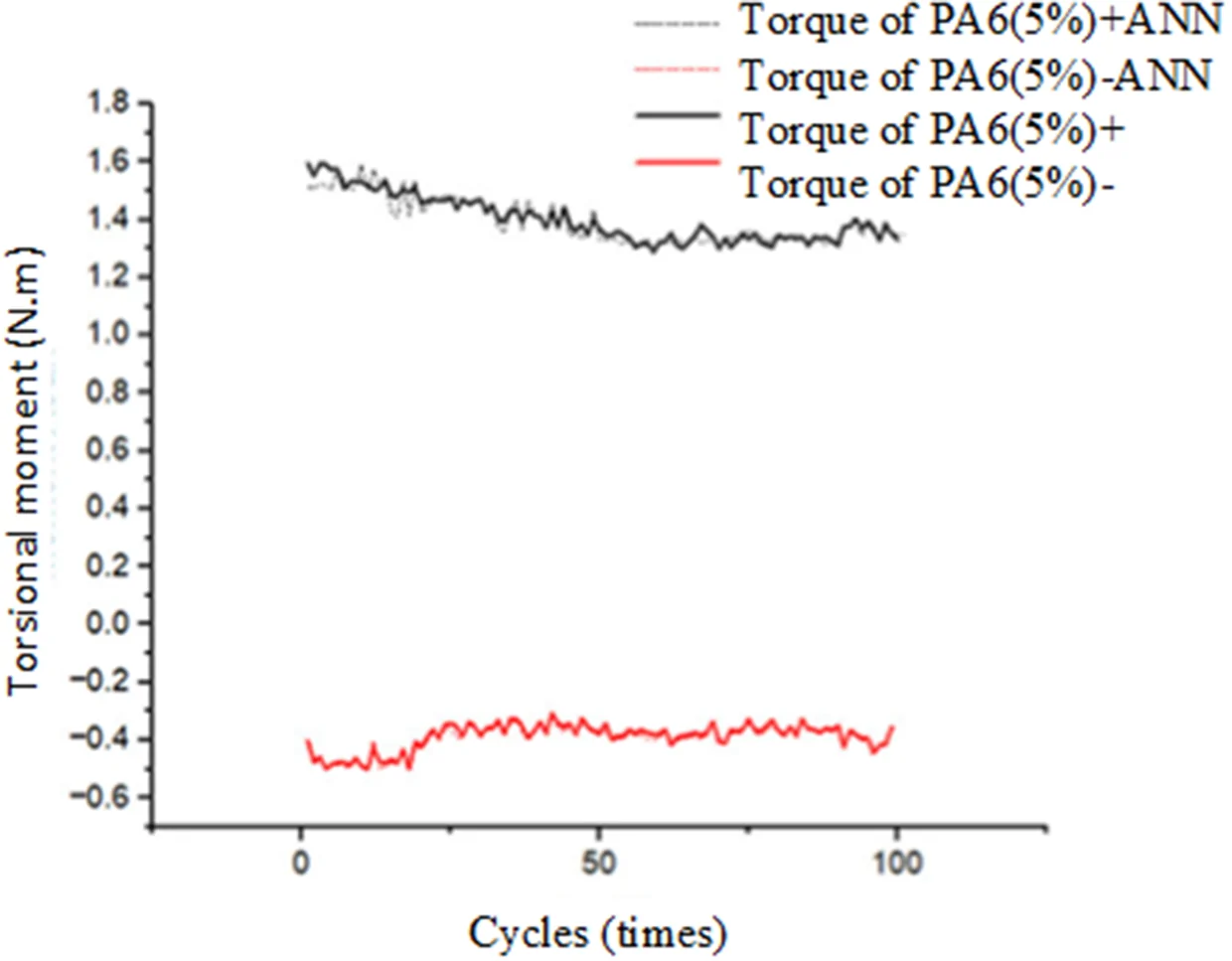
Comparison of experimental data and ANN model predictions for PA6 (5%).

**Fig 9:**
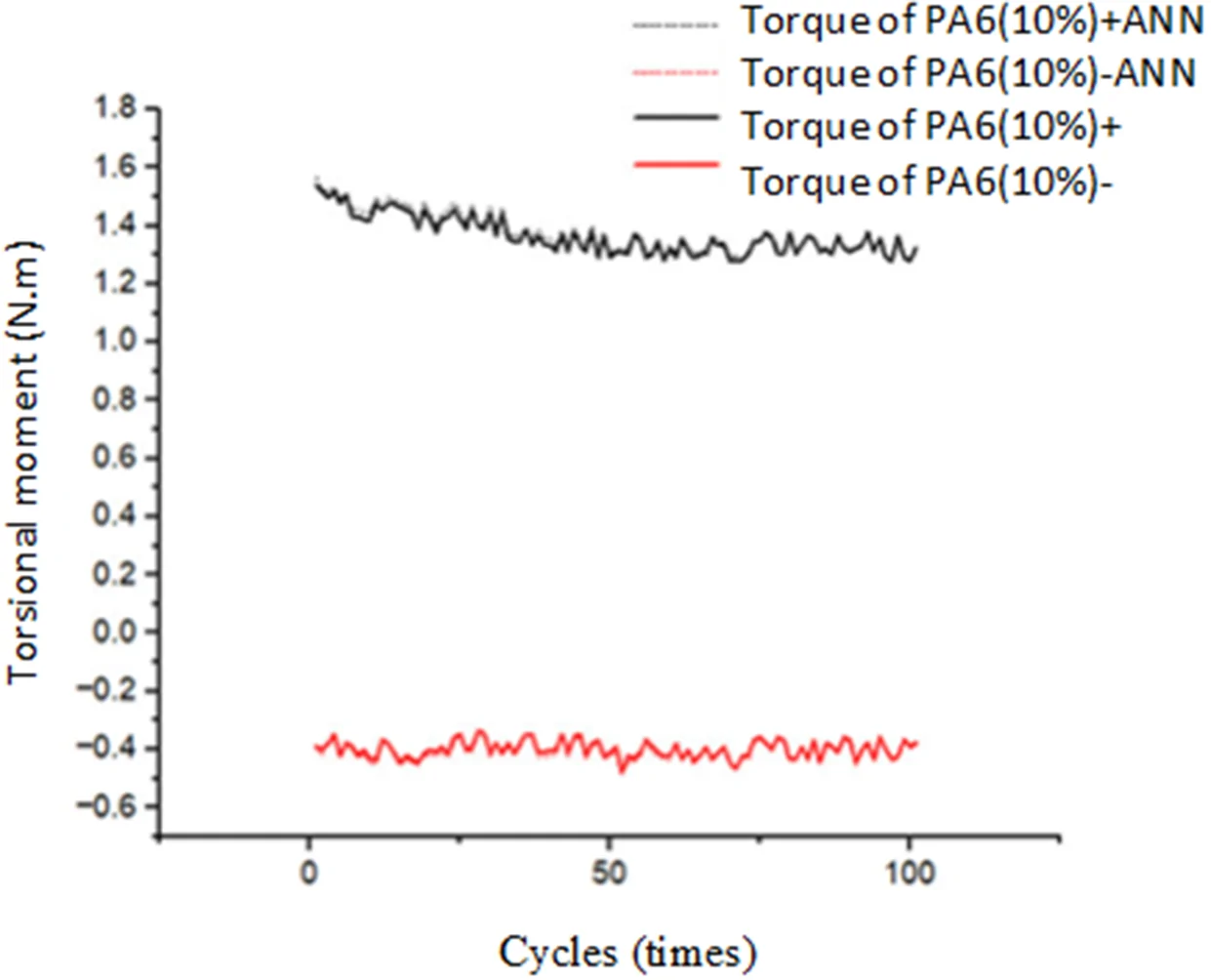
Comparison of experimental data and ANN predictions for PA6 (10%).

**Fig 10:**
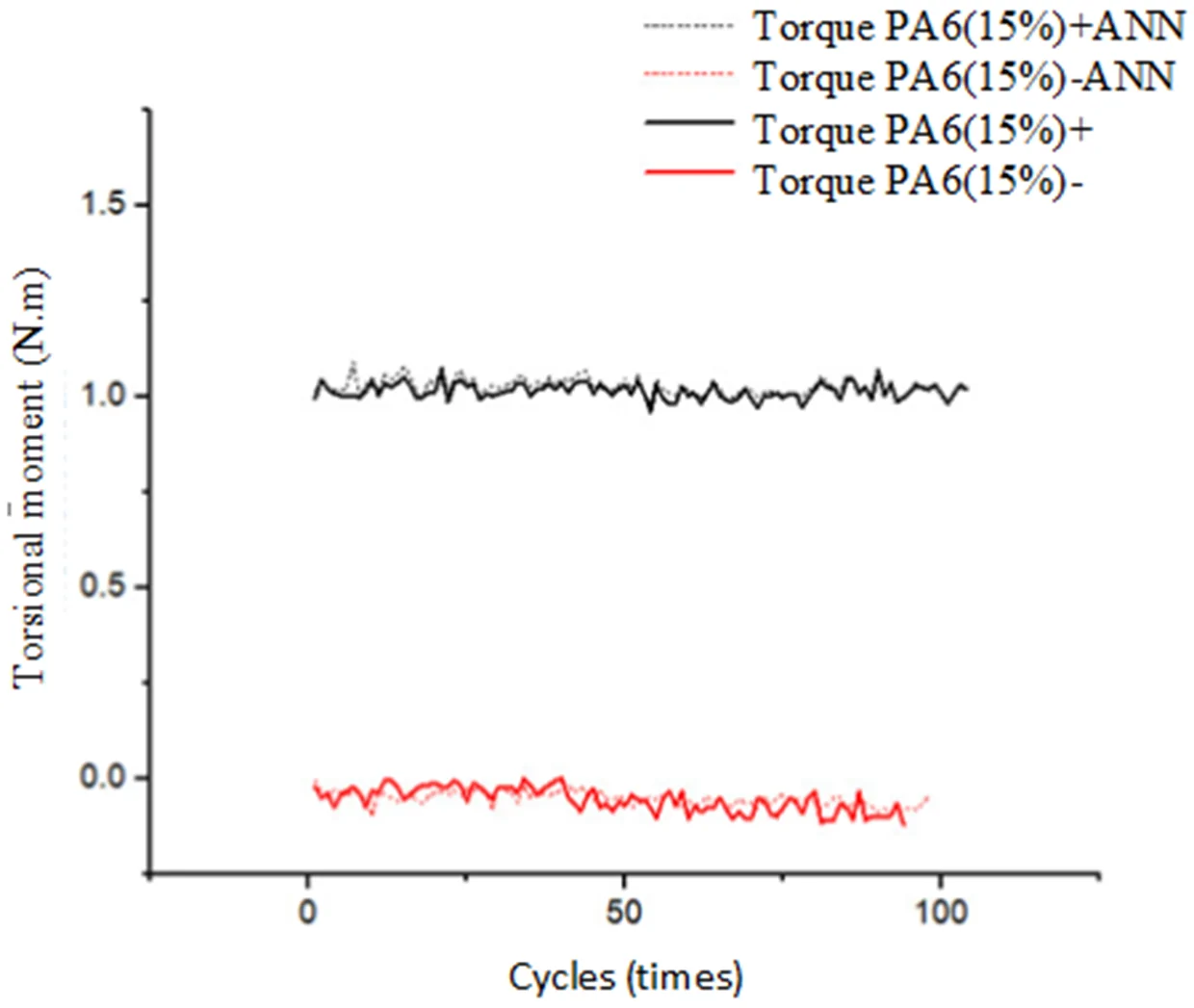
Comparison of experimental data and ANN model predictions for PA6 (15 wt. %).

**Fig 11:**
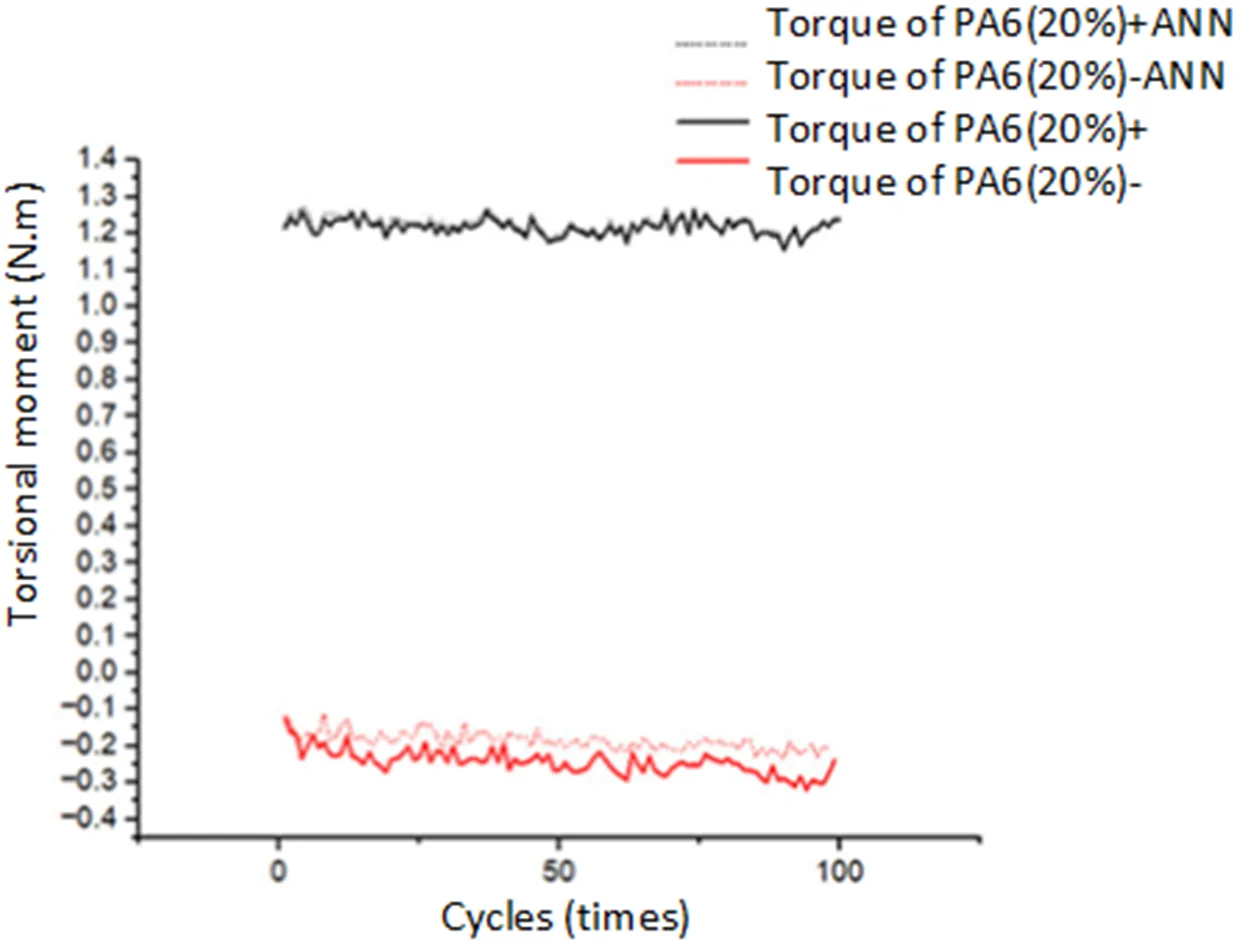
Comparison of experimental data and ANN model predictions for PA6 (20%).

**Fig 12:**
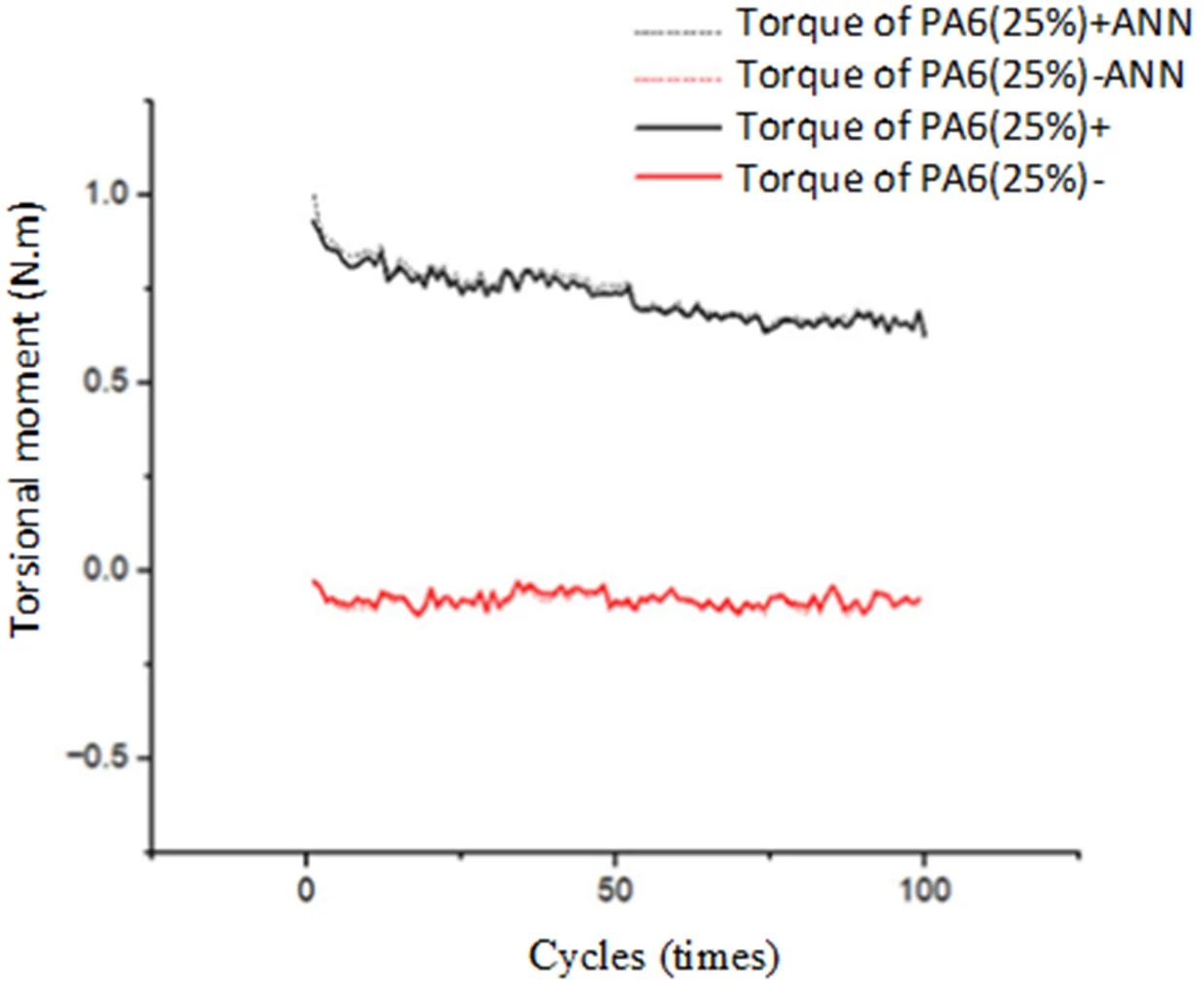
Comparison of experimental data and ANN model predictions for PA6 (25%).

**Fig 13:**
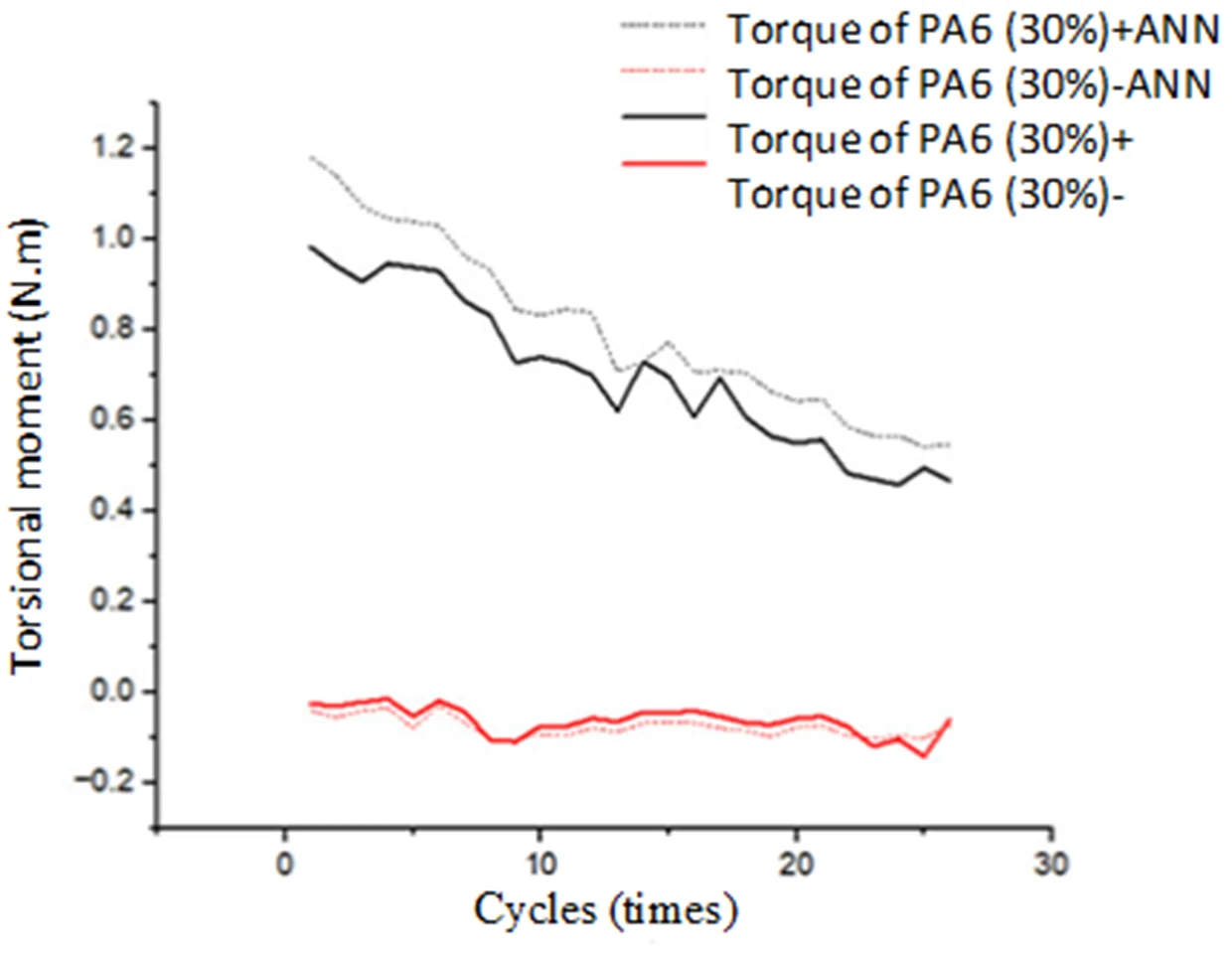
Comparison of experimental data and ANN model predictions for PA6 (30%).

The Fig 5 illustrates the variation of torsional moment with respect to the number of loading cycles for ABS. The horizontal axis represents the number of cycles, while the vertical axis shows the torsional moment (N.m). The black curve (ABS+) starts at a relatively high value and gradually decreases as the cycles increase, indicating that the material initially has strong torsional resistance but undergoes progressive degradation due to fatigue damage accumulation. In contrast, the red curve (ABS-) remains at a much lower level with small fluctuations and shows a nearly stable trend over the entire range of cycles, suggesting lower torsional strength but more consistent behavior under repeated loading.

The Fig 6 presents the variation of torsional moment as a function of the number of loading cycles for PP material. The black curve (PP+) starts at a relatively high value and gradually decreases with increasing cycles, indicating that the material initially exhibits good torsional resistance but experiences progressive degradation due to fatigue. In contrast, the red curve (PP-) remains at a significantly lower level and fluctuates slightly around a nearly constant value, suggesting lower torsional strength but more stable behavior over repeated loading.

The Fig 7 shows the evolution of torsional moment with respect to the number of loading cycles for PA6 material. The black curve (PA6+) begins at a relatively high value and gradually decreases as the number of cycles increases, reflecting good initial torsional resistance followed by progressive degradation due to fatigue. Meanwhile, the red curve (PA6-) remains at a much lower level with small fluctuations and exhibits a relatively stable trend throughout the cycles, indicating lower torsional strength but more consistent behavior under repeated loading.

The Fig 8 illustrates the variation of torsional moment with respect to the number of loading cycles for PA6 reinforced with 5 wt.% glass fiber. The black curve (PA 5 wt.%) starts at a relatively high value and gradually decreases as the number of cycles increases, indicating strong initial torsional resistance followed by progressive degradation due to fatigue damage accumulation. In contrast, the red curve (PA5%-) remains at a much lower level with slight fluctuations and maintains a relatively stable trend throughout the cycles, suggesting lower torsional strength but more consistent behavior under repeated loading.

Fig 9 presents the torsional moment versus angular displacement curves of the injection-molded compliant mechanism component under cyclic torsional fatigue loading. The positive moment curve, starting at approximately 1.6 N.m, shows a gradual decline with increasing number of cycles, indicating progressive softening or fatigue degradation in the positive loading direction. In contrast, the negative moment remains relatively stable around −0.4 N.m throughout the test with only minor fluctuations. This asymmetric torsional behavior highlights the directional dependence of the component’s mechanical response, which is typical for compliant mechanisms with non-symmetric geometry. The results demonstrate the overall torsional fatigue performance of the component and provide valuable experimental data for validating the proposed ANN-based prediction model.

Fig 10 shows the torsional moment (moment xoắn) versus the number of cycles for different PA 15% material variants under cyclic torsional loading. The results indicate that the unmodified PA 15 wt.% specimen (red line) exhibited a significantly lower torsional moment, stabilizing around 0.1–0.2 throughout the test. In contrast, the PA 15 wt.%, PA 15 wt.% and PA15 wt.% + variants maintained considerably higher and more stable torsional moments, approximately in the range of 1.0–1.1, with only minor fluctuations even after 100 cycles. These findings demonstrate that the incorporation of Neural Network (NR), or the enhanced “+” formulation substantially improves the torsional load-bearing capacity and fatigue resistance of the PA15% material under repeated torsional stress.

Fig 11 presents the torsional moment as a function of the number of cycles for various PA6 20 wt.% material formulations under repeated torsional loading. The unmodified PA6 20 wt.% specimen (red line) displayed a markedly low torsional moment, remaining stable in the range of approximately 0.3–0.4 throughout the test. In sharp contrast, the PA6 20 wt.%, PA 20 wt.%, and PA6 20 wt.% variants exhibited significantly higher and more stable torsional moments, consistently around 1.2–1.3 with only minor fluctuations even after 100 cycles. These results clearly demonstrate that the incorporation of the enhanced formulations substantially improves the torsional load-bearing capacity and fatigue resistance of the PA 20 wt.% material compared to the baseline version.

Fig 12 illustrates the torsional moment versus the number of cycles for different PA 25 wt.% material variants under cyclic torsional loading. The unmodified PA 25 wt.% specimen (red line) maintained a consistently low torsional moment, stabilizing around 0.2 throughout the test duration. In contrast, the PA 25 wt.%+ and PA 25 wt.% + variants exhibited significantly higher initial torsional moments, starting near 1.0 and gradually decreasing to approximately 0.6–0.7 after 100 cycles. The results indicate that the incorporation of the enhanced formulations markedly improves the torsional load-bearing capacity of the PA25% material compared to the baseline, although a moderate decline in moment is observed with increasing cycle number, suggesting some degree of fatigue-induced softening.

The cyclic fatigue behavior of PA6 composites containing 30 wt.% glass fibers, is presented in Fig 13 through the change of torque as a function of the number of cycles. As shown in the Fig, a general decreasing tendency is noted for all the positive torque values of the composite samples. This indicates the mechanical degradation of the composite material under cyclic loading conditions. It is also noted that the composite material PA6 (30%) byANN presents the highest positive torque values at the beginning of the test (approximately 1.2 units) and retains the highest load-bearing capacity over the 25 cycles compared to the reference composite material PA6 (30 wt.%)+. Moreover, while the positive torque values show a major reduction for all the composite samples, the negative torque values (denoted by the red colors) remain almost constant at zero, revealing the asymmetric behavior of the composite material when loaded under bidirectional stress. Some minor fluctuations of the torque values are probably due to internal micro-structural changes of the composite material, such as the gradual debonding of glass fibers from the PA6 composite material during cyclic deformation.

The ANN models showed excellent agreement with the experiment results for all materials, achieving high accuracy (R^2^ ≥ 0.94). The ANN models for ABS, PP, and PA6 (0–30 wt.% GF) materials were created, using input variables such as injection molding parameters, glass fiber content, and cycle number, with torque as the output. The data was divided into training, testing, and validation sets (10 wt.%, 20 wt.%, and 70 wt.% of the data, respectively). The ANN model consisted of a multilayer perceptron structure with 2–3 hidden layers, using the Adam optimizer, mean square error loss, and early stopping criteria.

The ANN models for all materials showed excellent performance (R ≥ 0.94, typically R = 0.96–0.98), achieving low RMSE values (0.08–0.22 N.m) and MAE < 0.15 N.m (~3−8% error). The best performance was obtained for the PP and PA6 (0−20 wt.% GF) materials, while higher error values were obtained for the ABS material due to nonlinear material degradation, and for the PA6 material (30 wt.% GF) due to brittle failure. The ANN model’s performance was also verified using cross-validation, achieving high R values (0.93–0.97).

The experiment results showed excellent material performance for the PP material, achieving low torque, while the ABS material showed higher stiffness but also fatigue degradation. The PA6 material showed improved material performance, such as higher stiffness and longer durability, due to the presence of glass fibers. However, the PA6 material showed brittle failure for 30 wt.% GF, indicating the optimal range for glass fiber content, i.e., 15–25 wt.%.

The ANN model shows excellent nonlinear performance, capturing the nonlinear material response, and can be considered a reliable tool for fatigue analysis, thus facilitating the design optimization process.

## 4. Conclusions

An Artificial Neural Network (ANN) model was proposed to predict the variation of the torsional moment with respect to different types of materials, glass fiber content, injection molding parameters, and the number of loading cycles. The proposed ANN model was able to achieve high accuracy for the prediction of the torsional moment with a high value of the correlation coefficient R ≈ 0.94, which is within the range of 0.96 to 0.98 for most of the materials. This verifies the potential of ANN models for the reliable and efficient prediction of the nonlinear behavior of injection-molded compliant mechanisms.

Because the present work focuses ANN prediction rather than on experimental fatigue characterization so constitutive material modeling, the plastic strain evolution was not directly measured during testing.

In conclusion, the research work has provided important experimental results and a practical ANN-based prediction tool for the design and optimization of injection-molded compliant mechanisms that are exposed to repeated torsional loading conditions. The results of the research work have also provided a scientific basis for selecting the appropriate types of materials and injection molding parameters for the development of compliant components for use in robotics and precision machines.
